# Pharmacological inhibition of REV-ERB stimulates differentiation, inhibits turnover and reduces fibrosis in dystrophic muscle

**DOI:** 10.1038/s41598-017-17496-7

**Published:** 2017-12-07

**Authors:** Ryan D. Welch, Cyrielle Billon, Aurore-Cecile Valfort, Thomas P. Burris, Colin A. Flaveny

**Affiliations:** Department of Pharmacology and Physiology, Saint Louis University School of Medicine, Saint Louis, MO 63104 USA

## Abstract

Duchenne muscular dystrophy (DMD) is a debilitating X-linked disorder that is fatal. DMD patients lack the expression of the structural protein dystrophin caused by mutations within the *DMD* gene. The absence of functional dystrophin protein results in excessive damage from normal muscle use due to the compromised structural integrity of the dystrophin associated glycoprotein complex. As a result, DMD patients exhibit ongoing cycles of muscle destruction and regeneration that promote inflammation, fibrosis, mitochondrial dysfunction, satellite cell (SC) exhaustion and loss of skeletal and cardiac muscle function. The nuclear receptor REV-ERB suppresses myoblast differentiation and recently we have demonstrated that the REV-ERB antagonist, SR8278, stimulates muscle regeneration after acute injury. Therefore, we decided to explore whether the REV-ERB antagonist SR8278 could slow the progression of muscular dystrophy. In mdx mice SR8278 increased lean mass and muscle function, and decreased muscle fibrosis and muscle protein degradation. Interestingly, we also found that SR8278 increased the SC pool through stimulation of Notch and Wnt signaling. These results suggest that REV-ERB is a potent target for the treatment of DMD.

## Introduction

Duchenne’s muscular dystrophy (DMD) is a progressive, fatal muscle wasting disorder caused by mutations within the *dystrophin* gene that affects 1:5000–10000 boys^1^. Even with treatment DMD patients have an expected lifespan of around 25 years. Boys affected with DMD are ambulatory only until about the age of 12, and become wheelchair bound and dependent on mechanical ventilation for respiratory support^2^. Patients eventually succumb to either cardiac or respiratory failure^3^. Dystrophin is a subsarcolemmal protein and a key component of the dystrophin-associated glycoprotein complex (DAG) that has both mechanical stabilizing and signaling roles in mediating interactions between the cytoskeleton, membrane and extracellular matrix. Loss of full-length dystrophin severely compromises myofiber stability and disrupts calcium signaling. As a consequence, normal muscle contraction produces excessively high levels of intracellular muscle damage. This results in rapid turnover of muscle fibers, inflammation, mitochondrial dysfunction, reduction in satellite cell numbers, and fibrosis that collectively deplete functional muscle mass^4,5^.

DMD is treated clinically with glucocorticoids, which slow loss of motor function and muscle turnover, possibly by inhibiting fibrosis^6,7^. However, the associated side effects of glucocorticoid use are often at odds with their beneficial effects in DMD. Physical therapy also has some proven therapeutic benefits, however, like glucocorticoids physical therapy cannot completely stymy DMD progression^7^. Other targeted approaches using anti-inflammatory agents that block NF-κB signaling have not advanced past clinical trials^8^. Cell based approaches that either employ CRISPR/Cas9 genome editing technology to repair DMD mutations and reprogram SC are still experimental^9,10^. More radical cell replacement therapies like autologous bone marrow transplant have not been comprehensively tested as clinically viable treatments^11^. Similarly inuitively appealing methods; exon skipping therapies, that attempt to rescue dystrophin protein expression in skeletal muscle (SkM) have been tested in patients^12–14^. These treatments utilize antisense oligonucleotides (AONs) to modify mRNA splicing of dystrophin transcripts and can potentially yield therapeutic benefits in about 83% of DMD patients. One AON, eteplirsen (or exondys 51), a morpholino designed to restore partial dystrophin expression by skipping exon 51, has successfully gained FDA approval. Yet in clinical trials involving eteplirsen the correlation between measured therapeutic outcomes and the degree of rescued dystrophin expression was unexpectedly inconsistent^15^. Inefficiencies of delivery and poor pharmacokinetics are some of the confounding technical challenges that are yet to be fully overcome with these agents. Meanwhile, efforts to eliminate incongruences in patient sorting and the manner in which therapeutic effects are measured in clinical trials should allow the therapeutic benefits of AONs to be more accurately gauged. The small molecule drug ataluren, which promotes ribosomal skipping of premature stop codons has also similarly been successfully used to rescue functional dystrophin expression and muscle function in DMD. Still, ataluren has only displayed therapeutic effects in select patient subgroups^16–19^. While all these treatments hold significant promise, they are still essentially palliative as they only delay loss of ambulation. Nevertheless, pharmacological therapies that have a favorable safety profile, potently combat muscle wasting and inhibit fibrosis with comparable efficacy to that of glucocorticoids are still much needed.

The nuclear receptor REV-ERB (REV-ERBα and REV-ERBβ) are ligand activated transcription factors and constituent transcriptional repressors^20^ encoded by the genes *NR1D1* and *NR1D2* respectively. REV-ERB is highly expressed in SkM and is an important regulatory component of the circadian clock, and a regulator of metabolism and cellular differentiation^21^. The circadian clock activator Brain and Muscle ARNT-Like 1 (BMAL1) is known inducer of myogenic and Wnt signaling in SkM and regulates adult SkM repair^22^. *Bmal1* expression has also been linked to SkM myosin heavy chain content and shown to be critical to the maintenance of the Pax7^+^ satellite cell population^23,24^. REV-ERB directly inhibits *Bmal1* expression by interacting with REV-ERB response elements (ROREs) at target gene promoters^25^. Interestingly, REV-ERB has been shown to inhibit myoblast differentiation by repressing myogenic signaling and cell cycle checkpoint regulators through an RORE-binding independent mechanism^26^. We have also shown that targeted disruption of REV-ERB activity stimulated muscle regeneration in response to acute injury^26^, suggesting that REV-ERB antagonists may be therapeutically beneficial in an assortment of myopathies.

We therefore explored whether disrupting REV-ERB activity using the REV-ERB antagonist SR8278 could slow the progression of muscular dystrophy in a model of DMD, the *mdx* mouse. REV-ERB antagonism improved muscle function, reduced fibrosis, stimulated mitochondrial biogenesis and induced an oxidative fiber phenotype in dystrophic mice possibly through activation of Wnt signaling. These observations suggest that pharmacologically active REV-ERB ligands should be capable of ameliorating the progression of dystrophinopathies.

## Results

### REV-ERB antagonism increases lean mass and improves muscle function in mdx mice

**Figure 1:**
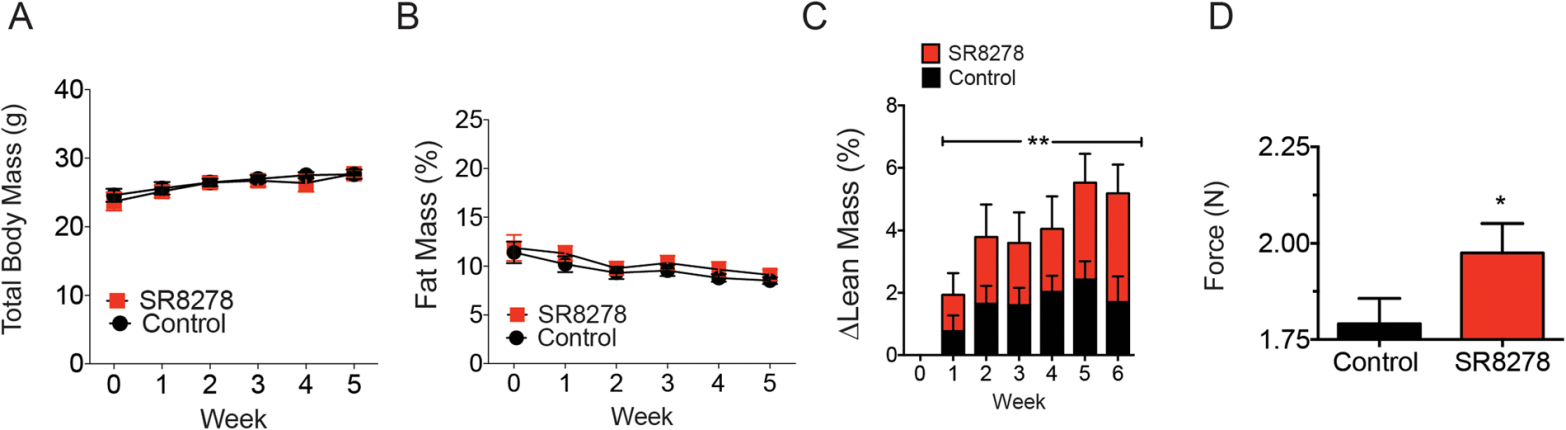
The Rev-Erb antagonist SR8278 increases lean mass and improves muscle function in dystrophic mice. (**A**) Total body mass of mdx mice treated with SR8278 or vehicle control over 6 weeks (n = 10). (**B**) Percent fat mass in mdx mice from (A). (**C**) Change in lean mass in mice treated with SR8278 or vehicle control **p < 0.05 data was analyzed using two-way ANOVA Data represented as mean and ± s.e.m. (**D**) Measured limb grip strength of mice treated with SR8278 or vehicle after 6 weeks. *p < 0.05 data was analyzed using student’s t-test. Data represented as mean and ± s.e.m.

REV-ERB is an inhibitor of myogenesis in myoblasts and pharmacological antagonism of REV-ERB stimulates muscle regeneration during acute injury^27–29^. Therefore, we postulated that inhibiting REV-ERB pharmacologically in a model of muscular dystrophy could yield some therapeutic benefit. We treated 10-week old mdx mice (n = 10 per group) with SR8278 or vehicle control for 6 weeks and assessed total body weight, adiposity and lean mass. Interestingly, SR8278 treatment increased lean mass and may have improved limb muscle function, as evidenced by grip strength assessment, without an overall increase in total mass (Fig. 1A–D). In order to probe the toxicity profile of SR8278 we performed clinical chemistry on the plasma samples of treated mice. Mice receiving SR8278 showed no elevation in the hepatic toxicity markers alanine aminotransferase (ALT), alkaline phosphatase (ALP) and urea suggesting that SR8278 was well tolerated and had no hepatotoxic effects (Supplementary Fig. 1A). There was similarly no evidence of SR8278 hepatomegaly in wild type C57Bl6J mice dosed with SR8278 for 3 weeks (Supplementary Fig. 1B). These observations suggest that disrupting REV-ERB activity may have systemically stabilized SkM in dystrophic mice without overt toxicity.

### Antagonizing REV-ERB improves muscle architecture and reduces fibrosis in dystrophic muscle

**Figure 2:**
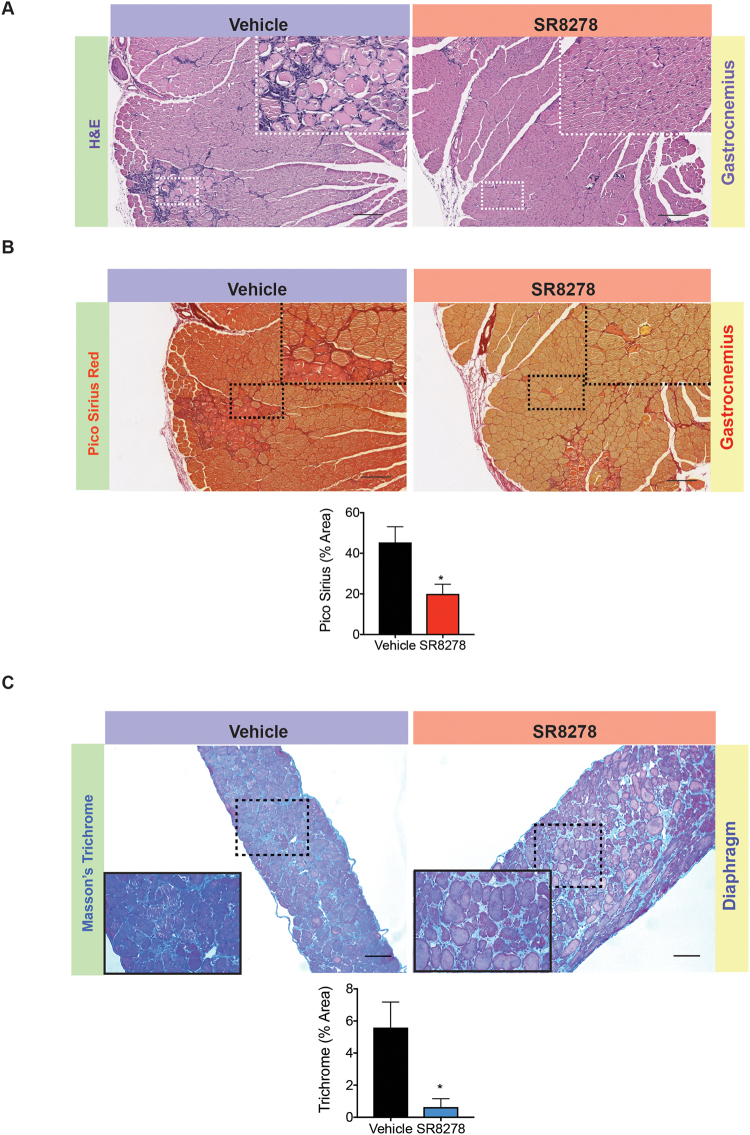
Antagonizing Rev-Erb improves muscle pathology and reduces fibrosis in dystrophic muscle. (**A**) Hematoxylin and eosin (H&E) staining of the gastrocnemius muscle in SR8278 and vehicle treated mdx mice improves structure and decreases fibrosis in dystrophic SkM. (**B**) Picrosirius red staining showing intramuscular fibrosis in the gastrocnemius of mice treated with SR8278 or vehicle control. Lower panel: graph showing percentage area staining positive for fibrous deposits in assayed muscles from SR8278 and vehicle treated mice. (**C**) Trichrome staining showing fibrous deposits in the thoracic diaphragm from mdx mice treated with SR8278 or vehicle. Picrosirius and trichrome staining were quantified using ImageJ as described previously^6^ (n = 6). (**A**–**C**) are Representative images from n = 6 mice per group. p < 0.05, **p < 0.01, ***p < 0.001. Statistical significance determined by two-tailed student’s t-test. Data represented as mean ± s.e.m.

In order to further understand the global effects of REV-ERB antagonism in SR8278 treated mice we performed histological assessment of the gastrocnemius and thoracic diaphragm muscles. H&E staining of mdx mouse muscle showed significant improvements in muscle fiber architecture with reduced basophilic infiltrates in mice treated with SR8278 (Fig. 2A). In addition, SkM of mice dosed with SR8278, exhibited reduced picrosirius red staining for fibrosis compared to vehicle treated mice (Fig. 2B). Similarly, fibrosis in the thoracic diaphragm, a major contributor to dystrophic patient mortality, was also significantly reduced in SR8278 treated mice (Fig. 2C). These observations indicate that SR8278 reduced intramuscular inflammation, and muscle turnover, and slowed the replacement of myofibers with fibrotic tissue; all key factors that drive DMD pathology.

### Pro-myogenic factor expression is induced by REV-ERB antagonism

**Figure 3:**
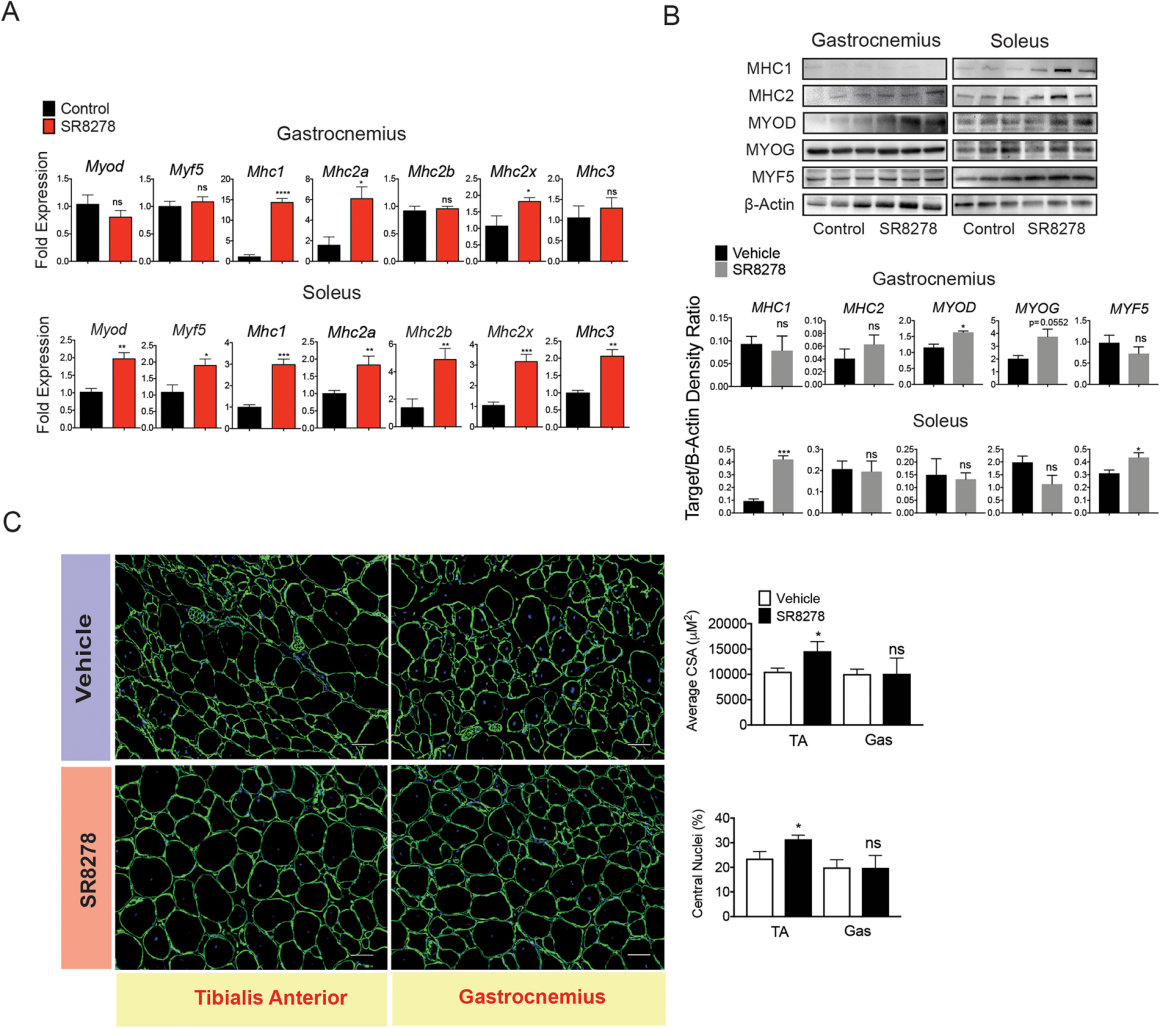
SR8278 increases pro-myogenic gene expression in dystrophic muscle (**A**). RT-QPCR showing expression of pro-myogenic factors *Myod*, *Myf5*, *Mhc1*, *Mhc2b*, *Mhc2x* and *Mhc3* in the soleus and gastrocnemius muscle of mdx mice in response to SR8278 or vehicle treatment for 6-weeks (n = 6) *p < 0.05 **p < 0.01 ***p < 0.001. Statistical significance determined using an unpaired two-tailed students t-test. Data represented as mean ± s.e.m. (**B**) Immunoblot showing expression of MHC1, MHC2, MYOG and MYF5 protein in the gastrocnemius and soleus of mdx mice treated with SR8278 or vehicle (n = 4). (**C**) Immunofluorescent staining of laminin protein in tibialis and gastrocnemius muscle sections from vehicle and SR8278 treated mice. Graphs show the measured cross sectional area and number of centralized nuclei in muscle sections from vehicle and SR8278 treated mice. *p < 0.05, **p < 0.01, ***p < 0.001. Statistical significance determined by two-tailed student’s t-test. (n = 6) Data represented as mean ± s.e.m.

Quantification of myogenesis expression in the muscle of mdx mice revealed that the myogenic factors myosin D (*Myod*), myogenic factor 5 (*Myf5*), myosin heavy chain 1 (*Mhc1*) myosin heavy chain 2b (*Mhc2b*) and embryonic myosin heavy chain (*Mhc3*) were all increased in antagonist treated mdx muscle (Fig. 3A). This induction of myosin heavy chain expression was specific to mdx muscle, as SR8278 failed to induce myosin heavy chain expression in wild type (C57Bl6J) mouse muscle (Supplementary Fig. 1C). These observations are in accord with that of previous studies where REV-ERB antagonists only mediated an effect on myosin gene expression under conditions where muscle turnover is actively occurring^26^. In parallel, immunoblot analysis showed an increase in MHC1, MHC2, MYOD and MYF5 protein expression in dystrophic mice treated with SR8278 (Fig. 3B). Importantly, SR8278 appeared to stimulate the expression of myosin heavy chain proteins specific to both slow oxidative (MHC1) and fast glycolytic (MHC2) muscle fibers (Fig. 3A,B). Interestingly, antagonist treatment did not alter muscle-specific MHC protein expression as the drug failed to significantly induce MHC1 expression in the gastrocnemius muscle, where it is not predominantly expressed, but stimulated both MHC1 and MHC2 expression in the soleus. With the observed increase in myosin heavy chain expression and stabilization in total lean mass considered, we decided to quantify the myofiber cross sectional areas in vehicle and SR8278 treated mice. Antagonist treatment marginally increased cross-sectional area and stimulated regeneration, evidenced by central nuclei, in the tibialis anterior specifically, but not the gastrocnemius (Fig. 3C) analogous to what has been observed previously in rodent models of acute muscle injury^26^.These results imply that antagonizing REV-ERB stimulates myogenic programs that enhance the expression of structural myosin heavy chains and may selectively stabilize lean mass in a muscle specific manner.

### REV-ERB antagonists promote mitochondrial biogenesis and oxidative fiber phenotype

**Figure 4:**
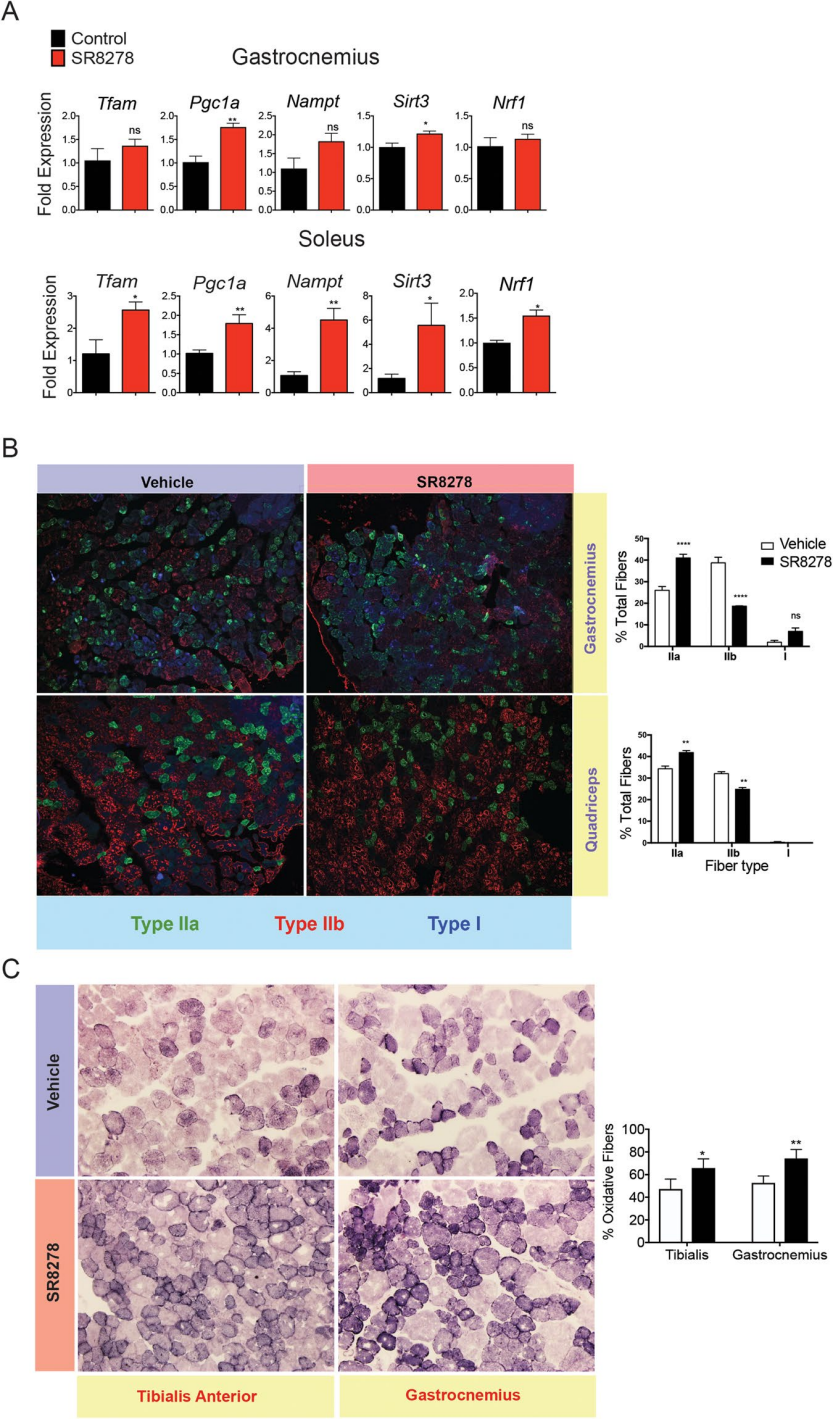
Rev-Erb antagonist treatment promotes mitochondrial biogenesis and an oxidative phenotype in dystrophic muscle. (**A**) Mitochondrial biogenesis gene expression- *Tfam*, *Pgc1a*, *Nampt*, *Sirt 3* and *Nrf1-* in the gastrocnemius and soleus muscle of SR8278 and vehicle treated mice (n = 10). (**B**) Immunofluorescent profiling of oxidative-type I and type IIa and glycolytic-type IIb fibers. *Right panel*-percentage composition of each fiber type in mouse muscle sections from vehicle and SR8278 treated mice (n = 6) (**C**) Histochemical staining for succinate dehydrogenase (SDH) enzyme expression in sections of the tibialis anterior and gastrocnemius muscle from vehicle or SR8278 treated mice. *Right panel * -percentage of oxidative fibres in Skm from vehicle and drug treated mice. (n = 6). p < 0.05, **p < 0.01, ***p < 0.001. Statistical significance determined by two-tailed student’s t-test. (n = 6) Data represented as mean ± s.e.m.

Mitochondrial biogenesis and activity are disregulated in DMD^30^. As a consequence, the oxidative capacity and prevalence of active oxidative fibers is reduced in dystrophic SkM. In order to further elucidate the mechanism through which SR8278 was improving muscle function and stabilizing lean mass we decided to assess the influence of REV-ERB antagonism on mitochondrial biogenesis and muscle fiber type. SR8278 treatment induced expression of a number of mitochondrial factors including *Tfam*, *Pgc1a*, *Nampt*, *Sirt3* and *Nrf1* in dystrophic soleus and gastrocnemius muscle (Fig. 4A), implying that mitochondrial biogenesis was enhanced by SR8278. Immunofluorescent fiber type profiling further revealed that SR8278 specifically increased the ratio of type IIa oxidative fibers relative to type IIb glycolytic fibers in SkM (Fig. 4B). In accord with this, the number of muscle fibers positive for succinate dehydrogenase (SDH) staining, a histological marker for oxidative muscle fibers, was also significantly increased by SR8278 treatment (Fig. 4C). Based on these analyses it appears that REV-ERB antagonism promoted mitochondrial biogenesis, and enhanced the oxidative fiber content and capacity of dystrophic muscle.

### REV-ERB inhibition enhances Wnt and Notch signaling in SkM

**Figure 5:**
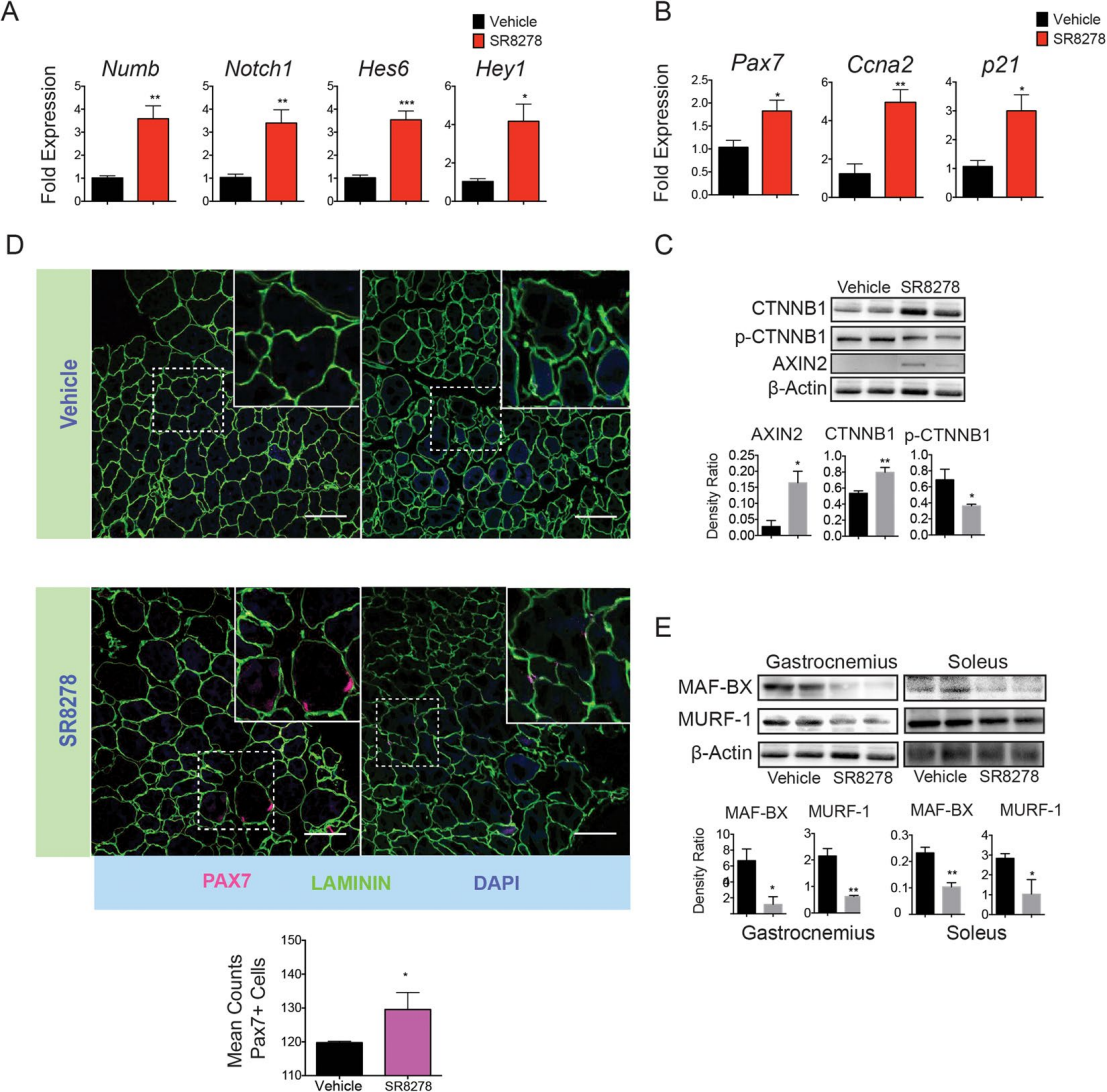
Rev-Erb disruption stimulates satellite cell activation in dystrophic muscle through activation of *Wnt* and *Notch* signaling. (**A**) mRNA expression of the *Notch* target genes *Numb*, *Notch1*, *Hes6* and *Hey1*. (**B**) mRNA expression of the SkM satellite marker *Pax7*, and the myoblast differentiation regulators *Ccna2* and *p21*, expression in (**A**) and (**B**) determined by RT-QPCR (n = 6). *p < 0.05, **p < 0.01. Statistical significance determined by two-tailed student’s t-test. (n = 6). Data represented as mean ± s.e.m. (**C**) Immunoblot showing the expression of the *Wnt* transcription factor β-catenin (CTNNB1) CK1 Ser37-phosphorylated β-catenin (p-CTNNB1) and the direct *Wnt* target gene *Axin2* in the gastrocnemius muscle of mdx mice treated with SR8278 or vehicle. Lower panel: quantified protein expression using density ratio analysis of protein expression. *p < 0.05 and **p < 0.01 statistical significance determined by two tailed-student’s t-test. (**D**) Immunofluorescence showing Pax7 expression in soleus muscle of mdx mice treated with SR8278 or vehicle. Lower panel quantified Pax7 + cells in the soleus muscle of mdx mice (n = 4). (**E**) Immunoblot showing expression of the Atrogin-1 complex proteins MAF-BX and MURF-1 in the soleus and gastrocnemius of SR8278 or vehicle treated mdx mice. Lower panel: quantified protein expression using density ration analysis. *p < 0.05 and **p < 0.01determined by two tailed-student t-test (n = 4). Data represented as mean and ± s.e.m.

Wnt and Notch control developmental signaling pathways that have key roles in muscle repair and satellite cell self-renewal. DMD patients display altered SkM Notch signaling that has been proven to be essential to satellite cell self-renewal and indispensable for the maintenance of SC quiescence^31–33^. During muscle repair, the Wnt transcription factor β-catenin (CTNNB1) mediates Wnt target-gene transcriptional regulation during satellite cell, proliferation, differentiation and fusion with myotubes^34–41^. Previous studies have also shown that activation of canonical Wnt signaling was therapeutic in rodent models of muscular dystrophy and acute injury^39,42–45^. We examined whether REV-ERB antagonism may also be modulating Notch and Wnt signaling activity in SkM. We discovered that SR8278 treatment increased both Notch (*Numb*, *Notch*) and Wnt target-gene expression (*Hes6*, *Hey1*) (Fig. 5A). This upregulation of Wnt and Notch target genes in mice receiving SR8278 correlated with enhanced expression of the cell cycle progression regulators *p21* and cyclin-dependent kinase A2 (*Ccna2*), which promote myoblast differentiation (Fig. 5B). Interestingly, Pax7 expression, a marker for SkM satellite cells, was also significantly upregulated in SR8278 treated muscle (Fig. 5B). SR8278 inhibited Ctnnb1 phosphorylation at Ser37 (Fig. 5C), a casein kinase 1 binding site that promotes Ctnnb1 proteasomal degradation and suppresses Wnt transcriptional activity^34,36^. SR8278 also promoted stabilization of total Ctnnb1 protein levels and increased protein expression of the Wnt target gene *Axin2* (Fig. 5C). Immunohistological assessment of Pax7 expression revealed an increase in the number Pax7^+^ cells along the myofibers margins in SR8278 treated mice (Fig. 5D). Collectively these observations suggest that antagonizing REV-ERB modulates Wnt and Notch signaling to increase Pax7^+^ populations in dystrophic muscle.

Another factor that influences muscle turnover, the ubiquitin-proteasome system in the SkM is involved in degrading sarcomeric proteins and myogenic response factors to promote muscle atrophy^46^. The muscle specific E3 ubiquitin ligases MURF-1 and MAF-BX tag proteins for 26 S proteasome mediated degradation and genetic ablation of these two factors protected mice from muscular atrophy^47^. In addition, MURF-1 and MAF-BX specifically are key mediators of the degradation of myosin heavy chains and MYOD^48^. We found that both MURF-1 and MAF-BX expression were downregulated by SR8278 treatment in soleus and gastrocnemius muscle (Fig. 5E). These findings suggest that REV-ERB inhibition slows myofiber protein turnover by reducing expression of MURF-1 and MAF-BX.

### Rev-Erb regulates Wnt signaling downstream of Wnt receptor activation in muscle cells

**Figure 6:**
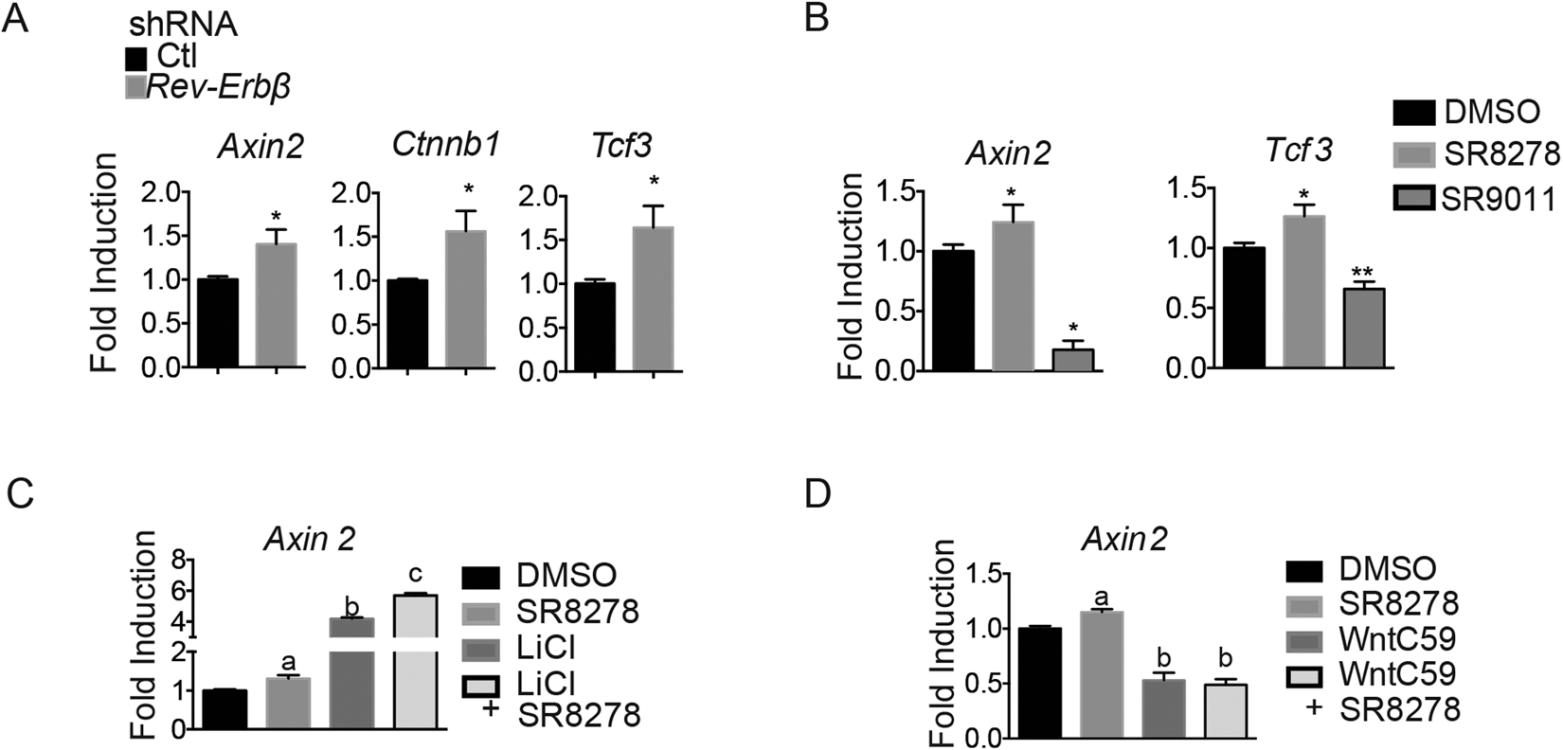
Rev-Erb regulates *Wnt* signaling downstream of Wnt receptor activation in muscle cells. (**A**) Expression of the Wnt target genes *Axin2*, *Ctnnb1* and *Tcf3* in response to Rev-Erb knockdown in cultured C2C12 myoblasts. (**B**) Expression of the Wnt target genes *Axin2*, *Tcf3* in C2C12 myoblast in response to the Rev-Erb antagonist SR8278 and agonist SR9011. (**C**) Expression *Axin2* in response to SR8278 or the Wnt agonist LiCl alone or in combination. (**D**) Induction of Axin2 expression in response to SR8278 or the Wnt inhibitor C59 alone or in combination. (n = 10) mRNA expression determined by RT-QPCR. *p < 0.05 and **p < 0.01 determined by two tailed-student t-test. Data represented as mean and ± s.e.m.

Based on the observed activation of Wnt in response to REV-ERB antagonism, we attempted to identify at which part of the Wnt pathway SR8278 may be mediating its effect. Firstly, we evaluated the effect of siRNA-mediated knockdown of REV-ERBα/β on Wnt transcriptional activity in differentiating C2C12 myoblasts. shRNA-mediated knock down of *REV-ERBβ* expression induced the Wnt signaling genes *Axin2*, *Ctnnb1*, and *Tcf3* (Fig. 6A). Secondly, we used the REV-ERB agonist SR9011 and antagonist SR8278 to elucidate whether Wnt target gene transcription was dependent on REV-ERB transcriptional activity. We observed that the REV-ERB agonist SR9011 and antagonist SR8278 reciprocally modulated Wnt activity in myoblasts (Fig. 6B). These observations coupled with that of mdx mouse muscle confirmed that REV-ERB expression and transcriptional suppression specifically inhibited Wnt signaling in myoblasts. To further probe this relationship between Wnt and REV-ERB we attempted to identify if REV-ERB’s influence on Wnt target gene expression could be enhanced by Wnt pathway activation and blunted by Wnt inhibition. We co-treated myoblasts with lithium chloride (LiCl), which disrupts CTNNB1 proteasomal degradation and enhances Wnt pathway activity and SR8278. As expected, SR8278 treatment and LiCl independently induced Wnt-target gene expression (*Axin2*) (Fig. 6C). In addition, co-treatment of myoblasts with SR8278 and LiCl had an additive effect on Wnt target gene expression (Fig. 6C). The Wnt inhibitor wnt-C59 prevents the palmitylation of Wnt ligands by Porcupine, Wnt ligand secretion and receptor mediated pathway activation^49^. To identify whether REV-ERB suppressed Wnt signaling upstream of Wnt receptor activation we assessed the combined effects of the Wnt inhibitor wnt-C59 on Wnt target gene expression in the presence or absence of SR8278. We observed that in cells treated with wnt-C59, SR8278 was unable to stimulate *Axin2* expression (Fig. 6D). These results support the concept that REV-ERB modulates Wnt signaling likely through an intracellular mechanism that is downstream of Wnt ligand-mediated activation.

## Discussion

Muscular dystrophy is a devastating muscle wasting disease that has no restorative clinical treatment. Previous work has shown that the circadian clock influences muscle repair and demonstrated the REV-ERB antagonists can be utilized to promote regenerative repair during acute injury^22,23,26,50,51^. This led us to test the therapeutic utility of a REV-ERB antagonist in a mouse muscular dystrophy model. Overall, we found that SR8278 effectively stabilized lean mass, improved SkM function, reduced fibrosis and stimulated SkM repair and mitochondrial biogenesis in dystrophic muscle.

Our investigation also revealed that mdx mice treated with SR8278 displayed lower intramuscular fibrosis. Fibrotic tissue deposits, the result of excessive buildup of extracellular matrix components into the bedding of muscle fibers, forms a mechanical barrier that restricts the formation of neuronal synapses and blocks myoblast expansion to repair damaged myofibers^52^. Pro-inflammatory M1 macrophages are one of the first innate immune cells to respond to muscle injury and are considered key to the healing process^53^. Conversely, anti-inflammatory M2 macrophages secrete a variety of growth factors that stimulate fibrogenesis. TGF-β is a well characterized factor that stimulates the switching of pro-inflammatory macrophages into the M2 anti-inflammatory phenotype^54^. Notably, in pre-brown adipocytes, REV-ERB can promote differentiation by suppressing the TGF-β/Smad2 pathway^55^. REV-ERB is also a well-characterized transcriptional regulator of macrophage activity^54,56,57^. It is however unknown whether TGF-β activity in macrophages is influenced by REV-ERB. Based on the inhibitory affect SR8278 had on intramuscular fibrosis, our observations suggest that SR8278 inhibition may be promoting pro-inflammatory M1 macrophage activity. However, whether REV-ERB can regulate muscle fibrosis by selecting for an M1 macrophage phenotype within the SkM microenvironment should be the subject of future investigations.

Muscle degradation is mediated by a collection of exogenous and endogenous factors that stimulate proteolysis. The E3 ubiquitin ligases MURF-1 and MAFbx tag myogenic factors and structural proteins for proteasomal degradation. Our investigations revealed that SR8278 decreased the expression of MURF-1 and MAF-BX in the soleus muscle. TNFα and IL-6 are both known myokines that induce muscle degradation by activating MURF-1 and MAF-BX. Previously it has been shown that the overexpression of *Rev-erbα* sensitized smooth muscle cells to TNFα and increased IL-6 expression^58^. It is not yet known if REV-ERBα or REV-ERBβ can induce myokine secretion, however, ablating CLOCK expression with siRNAs modulates myokine expression including IL-6 in human myotubes^59^. Further studies should be designed to determine if REV-ERB can modulate IL-6 secretion from myofibers.

Myogenic response factors are master regulators of SkM regeneration. We observed a robust increase of myogenic response factor expression, suggesting REV-ERB antagonism mediated muscle regeneration in mdx SkM. *Bmal1* expression, a direct REV-ERB target gene has been associated with the stimulation of muscle repair through activation of Wnt signaling. We speculated therefore that REV-ERB may also regulate Wnt signaling in SkM. Our results suggest that REV-ERB can regulate *Wnt* signaling by stabilizing CTNNB1 downstream of *Wnt* receptor activation. Whether cross talk between REV-ERB and *Wnt* signaling pathways in muscle is dependent on *Bmal* or other circadian factors remains to be determined.

In mdx muscle the SC population was also increased by pharmacological inhibition of REV-ERB. Investigations by Chaterjee *et al*. showed that *Bmal1* deficient mice displayed a reduction in Pax7^+^ SC through a yet to be described mechanism^22,23^. As REV-ERB is a repressor of *Bmal1* transcription, our results suggest that SR8278 may be inducing SC self-renewal through *Bmal1*. However, in this and other studies, the observed effects of REV-ERB antagonism are more pleiotropic than can be accounted for by BMAL1 and CLOCK regulated transcription and appear to be directed more by tissue specific factors such as Nuclear Factor-Y and Hepatocyte Nuclear Factor 6^26,60^. However, whether the circadian clock is integral to the process of REV-ERB directed muscle repair is unclear. RNA seq analysis of muscle samples from patients with collagen VI myopathies have linked aberrant circadian regulation to deregulation of autophagy genes, with the effect on collagen VI regulation mirrored in *Bmal1* knockout mice^61^. While the contributions of diurnal fluctuations to muscle turnover have been identified^22,23,62^ it is uncertain what circadian factors contribute to DMD dystrophinopathy.

Mitochondrial dysfunction, defined by lower energy capacity and high ROS production, are secondary physiological features of DMD that further exacerbate the dystrophic phenotype^63,64^. Activation of mitochondrial biogenesis and a switch to oxidative fiber types have been shown to help ameliorate dystrophic decline in mdx mouse models^65,66^. The effects of a REV-ERB antagonist on mitochondrial biogenesis and oxidative capacity were previously unknown. Here, SR8278 increased the expression of mitochondrial biogenesis genes and ushered a switch to enhanced oxidative fiber activity in dystrophic SkM. In contrast, previous studies investigating REV-ERB ligands in SkM found that activating the receptor increased oxidative gene expression and stimulated mitochondrial biogenesis in mouse muscle^50^. REV-ERB expression decreases during muscle differentiation and its expression has been shown to inhibit myotube formation^26,28^. Conversely, *Nr1d1* knockout mice have reduced skeletal muscle function and reduced lean mass. It has been demonstrated that REV-ERB promoter occupancy is distinct in differentiating and fully differentiated myoblasts^26^. The regulation of mitochondrial biogenesis and oxidative metabolism by REV-ERBs may therefore be dependent on the dynamics of REV-ERB promoter/enhancing binding in proliferating versus fully differentiated muscle. It is also important to note that muscle pathophysiology is also distinct in normal versus dystrophic mice. Dystrophic muscle undergoes rapid cycles of SkM destruction and repair that does not occur in normal differentiated muscle. Considering mitochondrial biogenesis is substantially elevated during muscle differentiation, it is possible that REV-ERB ligands may stimulate mitochondrial biogenesis through distinct or context specific mechanisms. Our results certainly affirm that REV-ERB antagonists enhance mitochondrial activity by inducing mitochondrial biogenesis factor expression. The underlying mechanisms that may drive the varied activity of REV-ERB and its ligands in different models remain undefined. Nevertheless, this investigation highlights that targeted inhibition of REV-ERB activity may be a highly effective approach for treating muscular dystrophies.

## Methods

### Mice

C57BL/10ScSn-Dmd^mdx^/J (mdx) mice were purchased from Jackson Laboratory (Bar Harbor, ME). All mice were housed in a 12h-12h light-dark cycle and received a standard chow diet. Mice were allowed food and water *ad libitum*. Mdx mice (n = 10) were dosed once a day by subcutaneous injections for 6-weeks. SR8278 (25 mg/kg) was dissolved in a 5:5:90 DMSO/cremophor/PBS vehicle. At the conclusion of the study mice were sacrificed by CO_2_ asphyxiation, followed by cervical dislocation. Mouse experimental procedures were approved by the Saint Louis University Institutional Animal Care and Use Committee (SLU-IACUC protocol#2474). All procedures were performed in accordance with the SLU-IACUC guidelines and regulations.

### Body Composition Analysis

Nuclear magnetic resonance (NMR) analyses were conducted once a week on mdx mice from 10-week old (n = 10 per group) until 16 weeks of age by utilizing a Bruker BioSpin LF50 Body Composition Analyzer (Bruker, Billerica, Massachusetts). Statistically significant differences in lean, fat and fluid mass were determined by 2-way ANOVA with an alpha set at 0.05.

### Grip Strength

Hind limb and forelimb grip strength was tested simultaneously with a digital force instrument (BIOSEB) as described previously^67^. At 16 weeks of age mdx mice (n = 10) were subjected to 3 grip strength tests with a minimum of 10-minute rest intervals between each test. To reduce user-specific bias, each blinded researcher (2 total) administered tests on two separate cohorts of mice. Measured values from these experiments were pooled and analyzed. Statistical significance between the respected groups was determined using an unpaired, two-tailed student’s t-test with an alpha set at 0.05.

### Blood Plasma Chemistry Analysis

For all *in vivo* experiments mouse blood samples were collected via cardiac puncture and isolated plasma was analyzed by the COBAS c311 system (Roche) assay kit for liver enzymes. Data was subjected to a student’s t-test.

### Immunohistochemistry

Muscle samples from mdx mice where frozen in OCT. Muscle samples were sectioned at 10 microns and blocked with 5% donkey, 1% BSA, 0.25% Tween20 for 30 minutes at room temperature. A rabbit polyclonal anti-Pax7 (dilution1:500; Abcam ab34360, Cambridge, UK) was incubated overnight at 4 °C in a humidity chamber. Secondary (anti-rabbit Cy3 1:100) was incubated for 1 hour in a humidity chamber at room temperature. The tissues were blocked again with 5% Rabbit serum in 1x PBS with 1% BSA, 2hrs in a humidity chamber at room temperature. Rabbit polyclonal anti-Laminin (dilution1:500; Sigma-Aldrich L9393, St. Louis, MO) was incubated overnight at 4 °C in a humidity chamber. Secondary AB (anti-Rb 488 1:300) was incubated at room temperature for an hour in a humidity chamber. The muscle tissue was then exposed to 4% paraformaldehyde for 10 minutes at room temperature. Prolong gold with DAPI was added and the slides were cover slipped. Pax7 + satellite cells where identified by co-localization with laminin. Relative Pax7 + satellite cell numbers where quantified by utilizing ImageJ software described previously^6^.

### Masson’s Trichrome

Diaphragm muscle samples were fixed overnight in 4% formalin and then embedded in paraffin. Muscle samples where sectioned and stained. Paraffin embedded muscle sections where stained using a Masson’s trichrome staining procedure. Staining was quantified using ImageJ as described previously^6^.

### H&E Staining

Muscle samples (gastrocnemius) were fixed overnight in 4% formalin and then embedded in paraffin. Paraffin embedded muscle sections where stained by a standard hematoxylin and eosin (H&E) protocol (Histowiz, Brooklyn NY). Slide images were taken by a Leica MC120 HD (Buffalo Grove, IL) attached to a Leica DM750 microscope (Buffalo Grove, IL).

### Picrosirius Red

Gastrocnemius samples were fixed overnight in 4% formalin and then embedded in paraffin. Paraffin embedded muscle sections where stained by a standard picrosirius red staining protocol (Histowiz, Brooklyn NY). Slide images were taken by a Leica MC120 HD (Buffalo Grove, IL) attached to a Leica DM750 microscope (Buffalo Grove, IL). Staining was quantified using ImageJ as described previously^6^.

### Succinate Dehydrogenase Staining

Fresh cryo-sections (10 μm) were incubated for 30 min at 37 °C in incubation medium (50 mM phosphate buffer, sodium succinate 13.5 mg/ml, NBT 10 mg/ml in water) placed in a Coplin Jar and then rinsed section in PBS. After staining, sections were fixed in 10% formalin-PBS solution for 5 minutes at room temperature and then rinsed in 15% alcohol for 5 minutes. Slides were mounted with an aqueous medium and sealed.

### Fiber Type Immunostaining

10 μm frozen sections were incubated with Mouse on Mouse IgG Blocking (Vector Lab) for 1 h at room temperature then washed 2 times in PBS. Sections were then incubated with a mixture of BA-D5 (Myosin Heavy Chain Type I), SC-71 (Myosin Heavy Chain Type IIa), BF-F3 (Myosin Heavy Chain Type IIb) (Developmental Studies Hybridoma Bank) for 45 min at 37 °C, in PBS-1%BSA. Sections were washed 3 times in PBS for 5 minutes at room temperature and then incubated with a mixture of the following secondary antibodies: anti-mouse IgG2b-Alexa405, IgG1-Alexa488, IgM-Alexa594 (ThermoFisher), all made in goat, diluted in PBS-1%BSA, for 30 minutes at 37 °C. Sections were washed for 5 minutes, 3 times with PBS at room temperature and mounted with ProLong Gold (Invitrogen).

### Quantitative PCR

Total RNA was isolated from muscle tissue by using the Trizol reagent (Invitrogen) and a PureLink RNA Mini Kit (Ambion) for C2C12 and human primary cells. Isolated RNA (1 μg) was reverse transcribed into cDNA by using qScript cDNA Synthesis Kit (Quanta Biosciences, Inc.). Primer sequences were designed using IDT primer design (Coralville, IA). Quantitative PCR (qPCR) was performed with SYBR Select Master Mix (Applied Biosystems) with select primers. Gene expression for mouse tissue and cells were normalized to *Gapdh*.

### Immunoblotting

Cells and muscle tissues were lysed using RIPA buffer with protease inhibitors. The extracted proteins were flash frozen and stored at −80 °C. Proteins were resolved by SDS-PAGE and transferred onto PVDF membranes (Bio-Rad). Membranes were blocked with 2% bovine serum albumin (BSA) for 1 hour at room temperature. Membranes were rinsed and incubated with primary antibodies over night at 4 °C. Primary antibodies utilized for immunostaining were rabbit monoclonal myosin heavy chain 2 (dilution 1:1000; Abcam ab91506, Cambridge, UK), mouse monoclonal myosin heavy chain 1 (dilution 1:2000; Abcam ab11083, Cambridge, UK), rabbit polyclonal anti-MURF-1 (dilution 1:1000, Abcam ab77577, Cambridge, UK), rabbit polyclonal anti-MAFbx (dilution 1:1000, Santa Cruz Biotechnology sc-33782, Dallas, Texas), rabbit polyclonal anti-Myod1 (dilution1:500; Abcam ab64159, Cambridge, UK), rabbit polyclonal anti-Myf5 (dilution 1:500, Santa Cruz Biotechnology sc-302, Dallas, Texas), mouse monoclonal anti-Myogenin (dilution 1:500, Santa Cruz Biotechnology sc-12732, Dallas, Texas), mouse monoclonal anti-Myosin Heavy Chain 3 (dilution 1:500, Santa Cruz Biotechnology sc-53091, Dallas, Texas), rabbit monoclonal anti-Axin2 (dilution 1:1000, Cell Signaling Technology 2151 S, Danvers, Massachusetts), rabbit polyclonal anti-p-βcatenin ser37 (dilution 1:1000, Santa Cruz Biotechnology sc-101651, Dallas, Texas), rabbit polyclonal anti-βcatenin (dilution 1:1000, Santa Cruz Biotechnology sc-1496R, Dallas, Texas), mouse monoclonal anti-β-actin HRP linked (dilution1:5000; Abcam ab20272, Cambridge, UK), rabbit polyclonal anti-PGC1α (dilution 1:200, Santa Cruz Biotechnology sc-1307, Dallas, Texas), and rabbit polyclonal anti-TFAM (dilution 1:500, Abcam ab131607, Cambridge, UK). Secondary HRP linked antibodies (dilution 1:3000, Abcam, Cambridge, UK) were incubated for 1 hour at room temperature. Chemiluminescence (Thermo Fisher Scientific) detection was conducted by MP ChemiDoc (Bio-Rad). The quantification of target protein was conducted in Image Lab software (Bio-Rad). The density ratios of target protein were normalized to β-actin levels.

### C2C12 Cell Culture

C2C12 cells were purchased from ATCC (Manassas, Virginia). Passage 3–7 C2C12 mouse myoblasts were proliferated in 15% fetal bovine serum in Gibco Dulbecco’s Modified Eagle Medium (DMEM) supplemented with GlutaMAX. Cells were incubated at 37 °C in a humidified atmosphere of 5% CO_2_. Cells were split when 50–60% confluent. Before differentiation, 90% confluent C2C12 cells were exposed to 5 μM of SR8278 or SR9011 for 24 hours in 15% FBS. Cells were then induced to differentiate by exchanging the proliferation media with fusion media (2% horse serum in Gibco DMEM). RNA extraction was conducted by lysing the cells with 350uL of Ambion Lysis buffer. Cells were frozen and mRNA extraction and purification conducted in accordance to protocol.

### Lentiviral Infection and Induction of shRNAs

SMARTvector Lentiviral Mouse Nr1d1 (or Nr1d2) mCMV-TurboGFP shRNA by Dharmacon RNAi Technologies (Lafayette, CO) were allowed to infect 15,000 proliferating C2C12 cells for 48 hours. Cells were split and seeded at a density of 15,000 in six-well plates. Twenty-four hours later cell selection was conducted by exposing the cells to 4 μg/mL of puromyocin. The shRNA mediated knock down of *Nr1d1* or *Nr1d2* in C2C12 cells was induced by doxycycline (1 μg/mL) exposure for 48 hours in select media (fusion or proliferation). Gene expression was utilized to assess targeted knock down when compared to a GFP expressing only control cell line.

### Wnt Modulators

After 48 hours of differentiating, cells where exposed to 5uM of SR8278 in a DMSO vehicle with or without the presence of LiCl (10 mM, Sigma Aldrich) or WntC59 (100uM, Bio-Techne) for 48 hours. The media with ligand and/or wnt modulators where replenished every 24 hours. The C2C12 cells were then lysed and their RNA isolated for future qPCR analysis.

### Data Availability Statement

All data generated or analyzed during this study are included in this published article (and its Supplementary Information files).

## Electronic supplementary material

Supplementary Figure
