# Supplementary material for: Pharmacological inhibition of REV-ERB stimulates differentiation, inhibits turnover and reduces fibrosis in dystrophic muscle
Source: Sci Rep. 2017 Dec 7;7:17142. doi: 10.1038/s41598-017-17496-7 (PMC5719458; doi:10.1038/s41598-017-17496-7)
Supplement: Supplementary file 1 — Supplementary Figure [file 41598_2017_17496_MOESM1_ESM.pdf]

**Pharmacological inhibition of REV-ERB stimulates differentiation, inhibits turnover and reduces fibrosis in dystrophic muscle**

**Authors:** Ryan D. Welch[1], Cyrielle Billon[1], Aurore-Cecile Valfort[1], Thomas P. Burris[1], and Colin A. Flaveny[1*]

[1]Department of Pharmacology and Physiology, Saint Louis University School of Medicine, Saint Louis, MO 63104.

[*]Corresponding author:

Colin A. Flaveny Ph.D. Email: colin.flaveny@health.slu.edu Phone: 314-977-6460

Supplementary Fig. 1

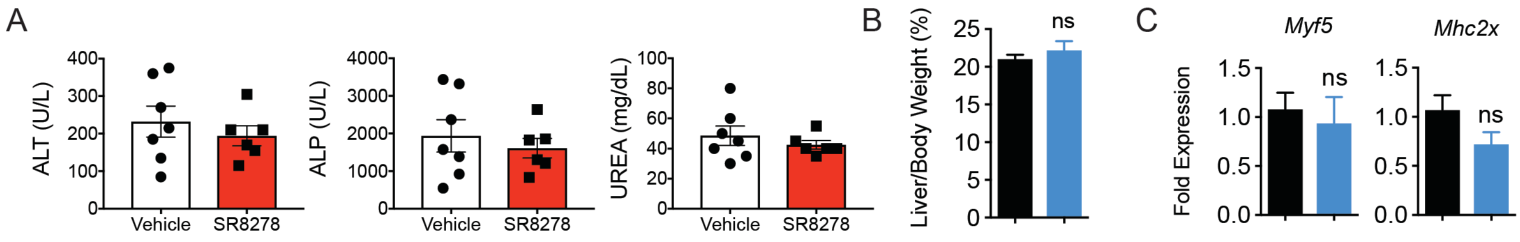


**Supplementary Fig. 1 A.** Clinical chemistry analysis of plasma levels of the liver toxicity markers, alanine amino-transferase (ALT), alkaline phosphatase (ALP) and urea. Isolated plasma was analyzed by the COBAS c311 system (Roche) assay kit for liver enzymes. n=10 for all experiments. *p<0.05 data was analyzed using student's t-test. Data represented as mean and ± s.e.m. **B.** Liver weight to body weight ratio of mice treated

with SR8278 or vehicle control. **C.** RT-QPCR showing expression of myogenic factors *Myf5* and *Mhc2x* in wild type mice treated with SR8278 or vehicle control
